# Profilin-1 is a negative regulator of mammary carcinoma aggressiveness

**DOI:** 10.1038/sj.bjc.6604038

**Published:** 2007-10-16

**Authors:** L Zou, M Jaramillo, D Whaley, A Wells, V Panchapakesa, T Das, P Roy

**Affiliations:** 1 Department of Bioengineering, University of Pittsburgh, Pittsburgh, PA, USA; 2 Department of Pathology, University of Pittsburgh, Pittsburgh, PA, USA; 3 Pittsburgh VA Medical Center, Pittsburgh, PA, USA

**Keywords:** profilin, breast cancer, cell migration, MDA-MB-231, HMEC, ligand interactions

## Abstract

Expression of profilin-1 (Pfn1) is downregulated in breast cancer cells, the functional significance of which is yet to be understood. To address this question, in this study we evaluated how perturbing Pfn1 affects motility and invasion of breast cancer cells. We show that loss of Pfn1 expression leads to enhanced motility and matrigel invasiveness of MDA-MB-231 breast cancer cells. Interestingly, silencing Pfn1 expression is associated with downregulation of both cell–cell and cell–matrix adhesions with concomitant increase in motility and dramatic scattering of normal human mammary epithelial cells. Thus, these data for the first time suggest that loss of Pfn1 expression may have significance in breast cancer progression. Consistent with these findings, even a moderate overexpression of Pfn1 induces actin stress-fibres, upregulates focal adhesion, and dramatically inhibits motility and matrigel invasiveness of MDA-MB-231 cells. Using mutants of Pfn1 that are defective in binding to either actin or proline-rich ligands, we further show that overexpressed Pfn1 must have a functional actin-binding site to suppress cell motility. Finally, animal experiments reveal that overexpression of Pfn1 suppresses orthotopic tumorigenicity and micro-metastasis of MDA-MB-231 cells in nude mice. These data imply that perturbing Pfn1 could be a good molecular strategy to limit the aggressiveness of breast cancer cells.

Cell migration, a fundamental aspect of tumour cell invasion and metastasis, involves dynamic remodelling of actin cytoskeleton via concerted actions of many different classes of actin-binding proteins (ABPs). Changes in actin cytoskeleton in malignant cells have been found to be correlated with altered expression of various ABPs (Vandekerckhove *et al*, 1990; Button *et al*, 1995; Wang *et al*, 1996; Clark *et al*, 2000; Pawlak and Helfman, 2001). Experimental manipulations restoring the normal expression levels of several different types of ABPs have been successful in suppressing the phenotype of transformed cells (Gluck *et al*, 1993; Tanaka *et al*, 1995; Nikolopoulos *et al*, 2000), therefore implying that deregulation of ABPs directly contribute to oncogene-induced transformed phenotype of tumour cells. In the emerging story on the role of ABPs in cancer, profilin-1 (Pfn1 – a ubiquitously expressed G-actin-binding protein) is found to be expressed at a significantly low level in both human breast cancer tissue and a variety of breast carcinoma cell lines (Janke *et al*, 2000), pancreatic (Gronborg *et al*, 2006) and hepatic (Wu *et al*, 2006) carcinoma cells compared to their normal counterparts. These interesting observations appear to suggest that loss of Pfn1 expression may have a general relevance in cancer progression. Pfn1-overexpressing CAL51 breast cancer cells failed to form tumours when xenografted subcutaneously in nude mice, which suggested that Pfn1 could also be a tumour-suppressor protein (Janke *et al*, 2000; Wittenmayer *et al*, 2004). Whether overexpression of Pfn1-elicits similar tumour-suppressive effect at the orthotopic site is not known.

Although Pfn1 was originally discovered as an actin monomer-sequestering protein (Karlsson *et al*, 1977), its cellular concentration does not appear to adequately account for the G-actin content in cells. Since Pfn1 enhances ADP-to-ATP exchange on G-actin, and is capable of adding ATP-bound G-actin to the barbed ends of growing filaments, it is now mainly thought to be a promoter of actin polymerisation (Witke, 2004). Besides actin, Pfn1 binds to phosphoinositides (mainly phosphatidylinositol-4,5-bisphosphate (PIP_2_) and phosphatidylinositol-3,4,5-triphosphate (PIP_3_) – Moens and Bagatolli, 2007) and a number of proline-rich proteins ranging from those participating in cytoskeletal to transcriptional control in cells (Witke, 2004). Pfn1-interacting protein families that are of particular relevance in the context of cell migration are the ones directly involved in cytoskeletal regulation, such as vasodilator stimulated phosphoprotein (VASP – Reinhard *et al*, 1995), Wiskott-Aldrich syndrome protein (WASP; example: Neuronal or N-WASP (Suetsugu *et al*, 1998), WAVE or WASP-associated verprolin homology (Miki *et al*, 1998)) and Diaphanous (example: mDia (Watanabe *et al*, 1997)). It has been hypothesised that these proline-rich proteins, when activated by signals, act as scaffolds to spatially recruit Pfn1–actin complex to the zones of active actin remodelling during cell migration.

Because of the multitude of its ligands, Pfn1 has been implicated in a wide range of cellular activities including proliferation, migration, endocytosis, mRNA splicing and gene transcription (Witke, 2004), which may explain why embryos null for Pfn1 or its homologue fail to survive in higher organisms (Verheyen and Cooley, 1994; Witke *et al*, 2001). A large number of pathogen-based model studies have implied Pfn1's function in actin-based protrusion during cell migration (Theriot *et al*, 1994; Mimuro *et al*, 2000; Grenklo *et al*, 2003). A more direct evidence of Pfn1's importance in mammalian cell migration, particularly for its role in cell protrusion, has been recently demonstrated in human vascular endothelial cells by our group (Ding *et al*, 2006). Given these findings, it is thus not clear why Pfn1 expression is significantly downregulated in different types of invasive cancer cells. This knowledge gap is partly due to lack of reports correlating Pfn1 expression with motility and invasiveness of tumour cells and/or their normal counterparts. We postulated that Pfn1 is a negative regulator of mammary carcinoma aggressiveness. Related to this postulate, we first investigated how loss of Pfn1 expression affects the motility of breast cancer cells and normal human mammary epithelial cells (HMEC). We then studied the effects of overexpression of either functional or ligand-binding deficient mutants of Pfn1 on breast cancer cell motility. To better relate to the process of tumour cell invasion *in vivo*, we next investigated whether perturbing Pfn1 expression modulates the intrinsic invasiveness of breast cancer cells. Finally, we evaluated the effect of Pfn1 overexpression on breast cancer cells *in vivo* in an orthotopic xenograft model system.

## MATERIALS AND METHODS

### Antibodies and reagents

Polyclonal Pfn1 and N-WASP antibodies were generous gifts of Drs Sally Zigmond (University of Pennsylvania, Philadelphia, PA, USA) and Dr Hideki Yamaguchi (Albert Einstein College of Medicine, NY, USA). Monoclonal VASP, vimentin, E-cadherin, keratin-18 and GFP antibodies were obtained from Pharmingen (San Diego, CA, USA). Polyclonal GFP antibody was purchased from the same source. Monoclonal GAPDH antibody was obtained from Abd-serotec (Raleigh, NC, USA). Polyclonal mDia antibody is from Abcam (Cambridge, MA, USA). Monoclonal actin and vinculin antibodies are from Chemicon (Temecula, CA, USA) and Sigma (St Louis, MO, USA), respectively. Collagen type I is a product of BD Biosciences (Bedford, MA, USA). All other cell culture reagents are products of Invitrogen (Carlsbad, CA, USA).

### Generation of Pfn1 constructs

The construction of GFP-Pfn1 expression vector has been previously described (Roy and Jacobson, 2004). We used PCR-based site directed mutagenesis to create H119E and H133S mutants of GFP-Pfn1. The forward and reverse PCR primers used for creating the H133S mutant were 5′-GTTATGAAATGGCCTCTAGCCTGCGGCGTTCCCA-3′ and 5′-TGGGAACGC CGCAGGCTAGAGGCCATTTCATAA-3′, respectively. The forward and reverse PCR primers for generating the H119E mutant were 5′-GCAAAGAAGGTGTCGAAGGTGGTTTG-3′ and 5′-CAAACCACCTTCGACACCTTCTTTGG-3′, respectively.

### Cell culture and transfection

HEK293 cells were cultured in DMEM-F12 media supplemented with 10% fetal bovine serum (FBS) and antibiotics. MDA-MB-231 breast cancer cells were cultured in EMEM media supplemented with 10% FBS, sodium pyruvate and antibiotics. Human mammary epithelial cells (source: Cambrex, Walkersville, MD, USA) were cultured in a complete growth media supplied by the manufacturer. Plasmid transfection of cells was perfomed using lipofectamine 2000 (Invitrogen) according to the manufacturer's protocol. Stable clones of MDA-MB-231 cells were selected and maintained using the regular growth media containing 1 mg ml^−1^ G418. In gene silencing experiments, cells were transfected with either a control siRNA or a custom-designed Pfn1-siRNA as previously described (Ding *et al*, 2006).

### Protein extraction and immunoprecipitation

Total cell lysate was prepared by extracting cells with modified RIPA buffer (50 mM Tris-HCl, pH 7.5, 150 mM NaCl, 1% NP-40, 0.25% sodium deoxycholate, 0.1% SDS, 2 mM EDTA, 50 mM NaF, 1 mM sodium pervanadate and protease inhibitors). For immunoprecipitation, 1000 *μ*g of lysate (extracted using a similar lysis buffer without SDS) was first precleared with 30 *μ*l of protein-G/protein A-conjugated agarose beads. Precleared lysate was incubated with 4 *μ*g of polyclonal GFP antibody for 4 h and then with 75 *μ*l of the same beads for an additional hour. Immunoprecipitated protein sample was washed three times with the lysis buffer, resuspended in 30 *μ*l of 2 × sample buffer and run on an SDS–PAGE. For immunoblotting, the antibodies were used at the following concentrations: Pfn1 (1 : 500), VASP (1 : 500), GAPDH (1 : 3000), vimentin (1 : 1000), actin (1 : 1000), N-WASP (1 : 1000), mDia1 (1 : 2500), vinculin (1 : 1000) and GFP (1 : 2000), keratin-18 (1 : 1000).

### Polyproline binding assay

Poly-L-proline (PLP, source: Sigma) was conjugated to cyanogens bromide-activated agarose beads as recommended by the manufacturer. Approximately, 150–200 *μ*g of cell lysates were incubated with 30 *μ*l of PLP beads on a rotating wheel for 1 h at 4°C. Poly-L-proline beads were pelleted by centrifugation, washed with the lysis buffer 3–4 times and resuspended in 2 × sample buffer for further immunoblot analyses.

### Biochemical fractionation

Cells were first washed with ice-cold F-actin stabilisation buffer (50 mM PIPES-pH 6.9, 50 mM NaCl, 5% glycerol, 5 mM EGTA, 5 mM MgCl_2_, 1 mM ATP, 1 mM DTT, 0.1% *β*-mercaptoethanol) and then extracted with buffer A (F-Actin stabilisation buffer supplemented with 0.5% Triton-X plus protease inhibitors) for 10 min to remove soluble proteins (contain G-actin). Culture plate was washed with buffer A and was further extracted with warm 1 × sample buffer to obtain the Triton-insoluble fraction.

### Immunostaining

For vinculin immunostaining, cells were washed three times with PBS, fixed with 3.7% formaldehyde for 15 min, permeablised with 0.5% Triton X-100 for 5 min and then blocked with 10% goat-serum for 30 min. After incubating with the primary antibody at 1 : 100 dilution for 1 h at room temperature, cells were washed five times with PBS and then incubated with a fluorophore-conjugated secondary antibody for an additional hour at room temperature. Stained cells were washed five times with PBS before mounting on slides for microscopy. For E-cadherin immunostaining, we used a similar procedure except in this case cells were fixed in ice-cold methanol for 10 min and blocked with 1% BSA for 45 min, before staining with the primary antibody at a 1 : 50 dilution. Phalloidin staining was performed as previously described (Roy and Jacobson, 2004).

### Cell motility and invasion assays

Wound-healing experiments were performed as previously described (Ding *et al*., 2006). Width of the wound for any field of observation was measured at 3–4 different locations. Wound closure was quantified by the change in wound width and averaged for three fields of observation per well from a 3–4 replicate set of samples for any experimental condition. Transwell migration experiments were performed as previously described (Roy and Jacobson, 2004) with slight modifications. Briefly, 50 000 serum-starved cells were seeded in triplicates in the upper chamber of the transwells (filters coated on both sides with 10 *μ*g ml^−1^ collagen) and allowed to migrate for a certain time (equal to 3 and 6 h for MDA-MB-231 cells and HMEC, respectively) towards the bottom chamber containing either serum-free (control wells) or serum-supplemented media. Nuclei of transmigrated cells (stained with Hoechst) were scored at 4–5 random fields of observation. To measure true chemotaxis, the average number of transmigrated cells in the control wells (represents random migration) was subtracted from the same achieved under the serum gradient. For single-cell motility assays, time-lapse imaging of cells was performed simultaneously at three random fields and at an interval of 60–90 s for a total duration of 90 min. The acquired images were analysed using the Metamorph software.

For invasion experiments, commercially available transwell chambers that are precoated with matrigel (Source: BD Biosciences, Bedford, MA, USA) were used. The invasion assay was performed according to the manufacturer's instructions where 25 000 cells were plated in 3–4 replicates in either invasion or control chambers (lacks matrigel layer) under a chemotactic gradient of serum. After 24 h, cells that have either trans-migrated (in control chambers) or invaded and reached the bottom of the filters were scored at five random fields of observation, and averaged for the replicate wells. Percentage of invading cells for any treatment group was calculated by dividing the average number of invading cells by that of trans-migrated cells. All motility and invasion experiments for gene silencing-based studies were carried out either 72 (for MDA-MB-231 cells) or 96 (for HMEC) h after siRNA transfection.

### Cell-adhesion assay

In total, 20 000 HMEC were non-enzymatically dissociated, stained with 5 *μ*M calcein AM (Invitrogen, Carlsbad, CA, USA) and then plated in triplicate on a dense monolayer of unlabelled cells in a 96-well plate. After allowing 30 min for cell–cell attachment, the culture plate was centrifuged at 200 **g** force for 2 min and then washed gently with PBS twice to remove the unattached cells. Cell–cell adhesion strength was scored by recording the fluorescence intensity of labelled cells that remained attached in the well using a plate fluorometer. Absolute fluorescence readings were background subtracted, averaged for the replicate set of samples from a total of four independent experiments for statistical comparison.

### Animal studies

Two million cells, suspended in 200 *μ*l of sterile PBS, were orthotopically injected into the right inguinal mammary fat pad of 4-week old nude female mice. After killing the animals at either 5–6- or 8-week time points after injection, tissues from lung, liver and spleen were harvested for histological staining. These experiments were performed in compliance with an approved protocol by the Institutional Animal Care Committee of the University of Pittsburgh.

### Statistics

All experimental data were analysed by ANOVA followed by Dunnett's *post hoc* test within a 95% significance level.

## RESULTS

### Loss of Pfn1 expression enhances motility of breast cancer and normal HMEC

**Figure 1:**
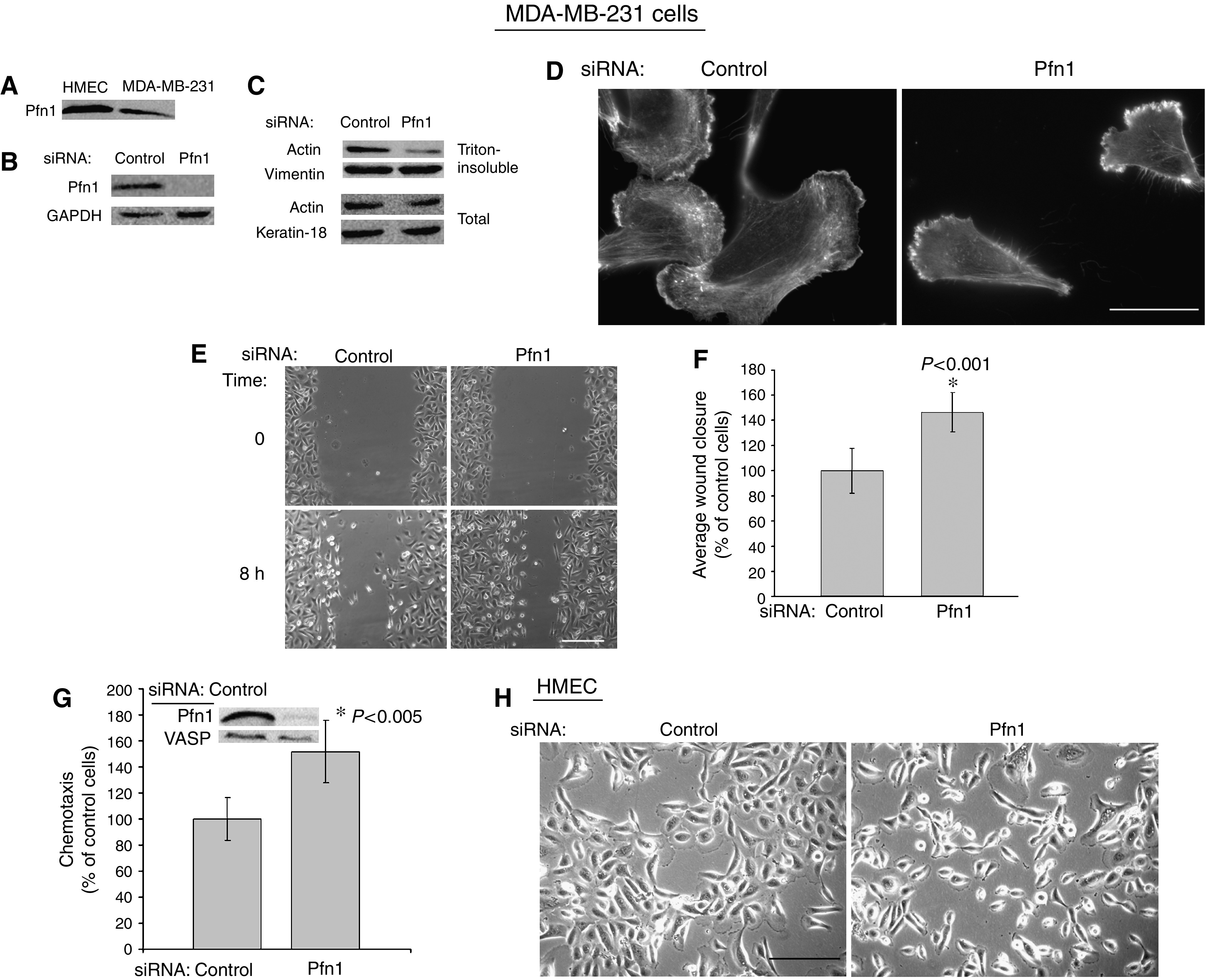
Silencing Pfn1 expression increases MDA-MB-231 and HMEC motility. (**A**) Immunoblot of total cell lysates (20 *μ*g loaded in each line) shows that MDA-MB-231 cells express significantly less Pfn1 compared to HMEC. (**B**) Immunoblot of MDA-MB-231 cell lysates confirms siRNA-mediated suppression of Pfn1 expression (the GAPDH blot serves as the loading control). (**C**) Immunoblot shows that Triton-insoluble fraction of MDA-MB-231 cell lysates has significantly less actin when Pfn1 expression is silenced (the vimentin blot serves as the loading control). Total actin expression is also slightly reduced in MDA-MB-231 cells after silencing Pfn1 expression (the keratin-18 blot serves as the loading control). (**D**) Phalloidin staining shows reduced flare of protrusion in Pfn1-depleted MDA-MB-231 cells (bar – 30 *μ*m). (**E**–**F**) Representative images of wound-healing experiments (**E**; bar – 200 *μ*m) and a bar graph (**F** *–* summarised from four experiments) illustrate the effect of silencing Pfn1 expression on MDA-MB-231 cell motility. (**G**) A bar graph summarises the results of two independent experiments that examined the effect of silencing Pfn1 on HMEC chemotaxis in a transwell-based assay (Pfn1 immunoblot in the inset demonstrates siRNA-mediated suppression of Pfn1 expression in HMEC with VASP blot serving as the loading control). (**H**) Phase-contrast images show dramatic scattering of Pfn1-depleted HMEC in culture (bar – 200 *μ*m).

Similar to previous observation of Pfn1 downregulation in human breast cancer tissue and a wide range of other breast carcinoma cell lines (Janke *et al*, 2000), we observed that MDA-MB-231, our model breast cancer cell line, also expresses significantly less Pfn1 compared to normal HMEC (Figure 1A). Because of a general theme of Pfn1 downregulation in many different breast cancer cell lines, we specifically hypothesised that downregulation of Pfn1 expression confers increasing aggressiveness to both normal HMEC and breast cancer cells. To test this hypothesis, we adopted gene-silencing approach where we transiently transfected MDA-MB-231 breast cancer cells and HMEC with either a control or Pfn1-specific siRNA, and examined the changes in cell motility. Figure 1B shows a Pfn1 immunoblot of MDA-MB-231 cell lysates confirming near 100% suppression of Pfn1 expression achieved within 72 h after siRNA transfection. Since Pfn1 can function as a promoter of actin polymerisation, particularly during cell protrusion, we asked whether loss of Pfn1 expression affects the actin cytoskeleton and morphology of MDA-MB-231 cells. Biochemical fractionation experiments revealed that cells lacking Pfn1 have significantly less actin in the Triton-insoluble fraction when compared to the same for control siRNA-treated cells, therefore meaning that Pfn1-depletion results in reduced F-actin content in MDA-MB-231 cells (Figure 1C). The overall actin level in MDA-MB-231 cells also appeared to be slightly reduced when Pfn1 expression was silenced (Figure 1C). We next performed phalloidin staining of transfected cells, which showed that although cells lacking Pfn1 develop spatial asymmetry marked by preferential protrusion at one end, the overall flare of lamellipodial protrusion and F-actin staining near the leading edge are markedly reduced with respect to the control cells (Figure 1D). The effect of silencing Pfn1 expression on cell motility was next analysed using a wound-healing assay, the results of which are depicted in Figure 1E–F. Data analyses revealed that suppressing Pfn1 expression leads to a significant 40% increase in motility of MDA-MB-231 cells (Figure 1F). These results were further confirmed by similar finding from transwell migration experiments (data not shown).

Similar to our finding in MDA-MB-231 cells, silencing Pfn1 expression in normal HMEC was found to decrease the cellular F-actin content by 35% (data not shown) and increase their chemotactic migration by a significant 51% in a transwell-based assay (Figure 1G – the immunoblots in the inset confirm siRNA-mediated suppression of Pfn1 expression in HMEC). Transwell data were further verified by similar observation from wound-healing experiments with HMEC (data not shown). Increased motility associated with loss of Pfn1 expression is also reflected by dramatic scattering (indicative of greater disseminative capacity) of Pfn1-siRNA-treated HMEC compared to control cells (displays typical clustered morphology) in subconfluent culture conditions (Figure 1H).

**Figure 2:**
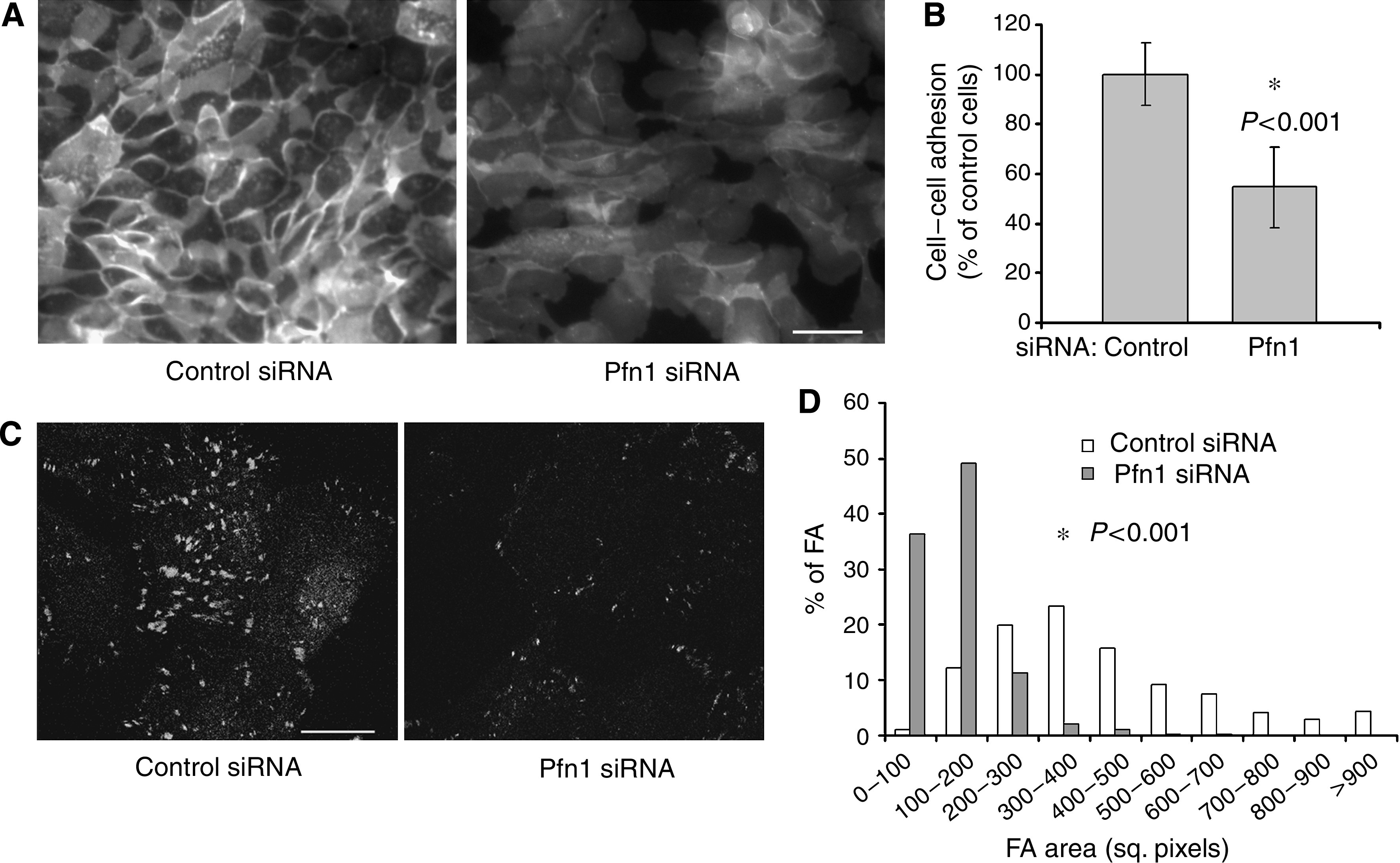
Loss of Pfn1 expression downregulates cell–cell and cell–cell matrix adhesions in HMEC. (**A**) Immunostaining shows reduced junctional distribution of E-cadherin in Pfn1-depleted HMEC compared to the control-siRNA-treated cells (bar – 50 *μ*m). (**B**) A bar graph illustrating the inhibitory effect of silencing Pfn1 expression on cell–cell adhesion (*N*=4 experiments). (**C**) Vinculin immunostaining shows less pronounced focal adhesions in Pfn1-depleted HMEC (bar – 24 *μ*m). (**D**) A histogram reveals the difference in the distribution of focal adhesion size between control and Pfn1-siRNA-treated HMEC.

Some of the hallmark changes that facilitate epithelial cell dissemination and migration during cancer progression are downregulation of both cadherin-based cell–cell and cell–matrix adhesions. Because of increased scattering of Pfn1-siRNA-treated cells in culture, we asked whether loss of Pfn1 expression is associated with altered adhesive properties of HMEC. Our immunostaining data showed that Pfn1-depleted HMEC have much less E-cadherin distribution at the cell–cell junctions compared to control siRNA-treated cells (Figure 2A). To further determine whether loss of Pfn1 expression actually inhibits HMEC to form cell–cell adhesion, we performed a functional cell-adhesion assay. Even within the first 30 min of cell attachment phase, there was a significant 46% reduction in the intercellular adhesion strength of HMEC when Pfn1 expression was suppressed (Figure 2B). Although centrifugation-based cell–cell adhesion assay as adopted in the present study is widely used, it suffers from a potential drawback that detachment of unlabelled cells from the underlying substratum, if occurs in response to centrifugal force, can confound the results. However, we did not see any evidence of disruption of monolayer formed by unlabelled cells in response to the applied centrifugal force for either of the treatment groups (data not shown), and thus we feel confident that our experimental readouts truly represent cell–cell adhesion strength. Also, since this assay scores the number of cells attached to a monolayer of cells as opposed to measuring the actual force, we preferred to obtain our experimental readout shortly after cell-seeding rather than at later time points where differences in the rate of cell proliferation between the two transfection conditions may confound the results. However, from a dramatic scattering of Pfn1-depleted HMEC in culture as shown in Figure 1H, a 46% reduction in experimental readout from the functional assay is most likely a gross underestimate of the actual downregulation of cell–cell adhesion induced by loss of Pfn1 expression. Next, to assess the effect of silencing Pfn1 expression on cell–matrix adhesions, we performed vinculin immunostaining of transfected cells which showed that HMEC lacking Pfn1 have much less pronounced focal adhesions (FAs) compared to control siRNA-treated cells (Figure 2C). A histogram in Figure 2D illustrates the significant difference in the FA size between the control (average size: 425±237 square pixels; *n*=775 FAs from 48 cells) and Pfn1-siRNA-treated cells (137±69; *n*=788 FAs from 51 cells). Taken together, these data demonstrate that loss of Pfn1 expression primes HMEC and breast cancer cells to a more migratory phenotype, and thus support our overall postulate.

### Overexpression of Pfn1 alters morphology and dissemination of MDA-MB-231 cells

**Figure 3:**
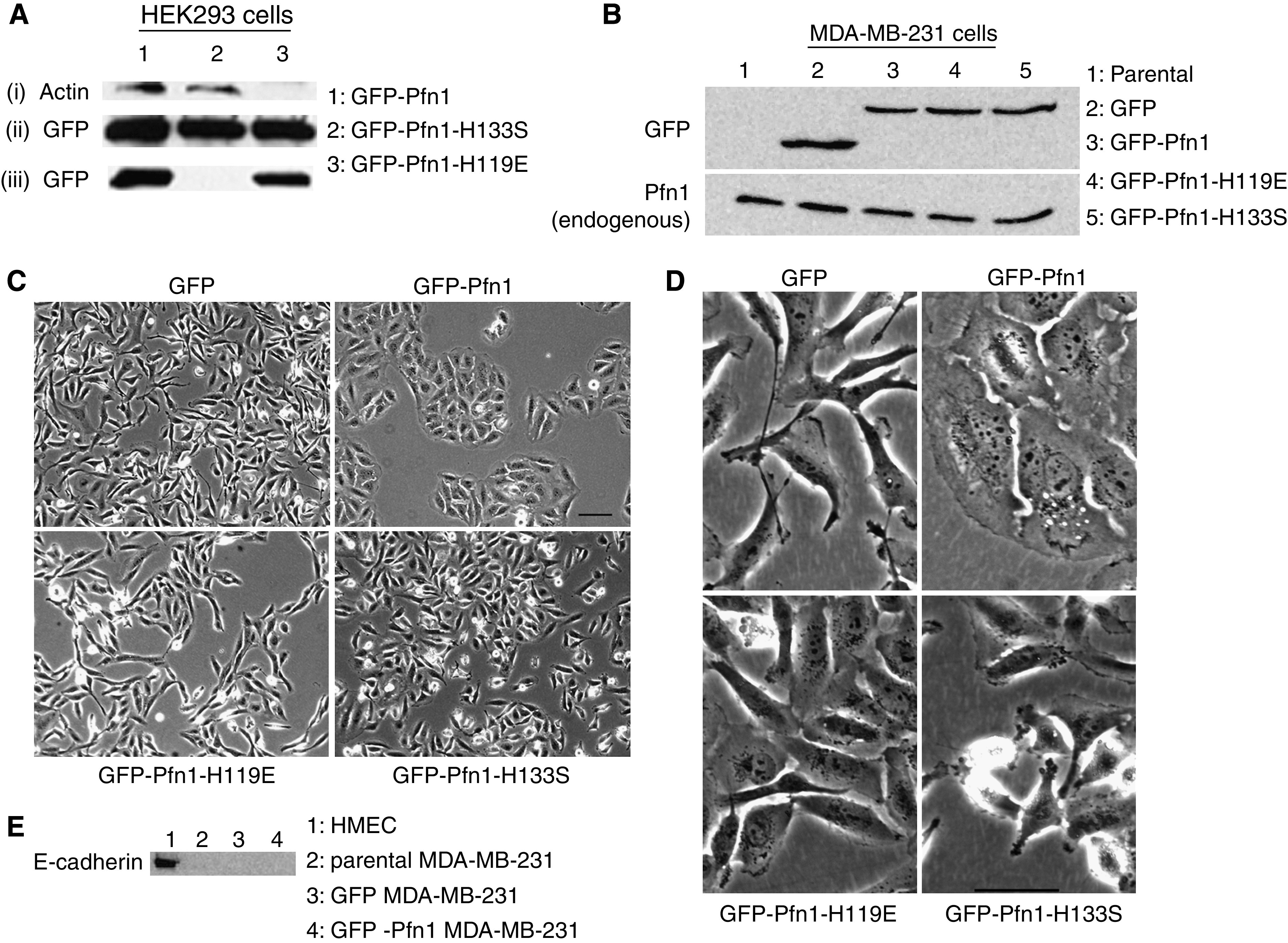
Overexpression of Pfn1 and its functional mutants. (**A**) Immunoprecipitated GFP-Pfn1 and its mutants (H119E, H133S) from HEK293 lysates probed with actin-antibody shows loss of actin co-precipitation with GFP-Pfn1-H119E (i). Reprobing the same blot with GFP antibody shows equal loading of immunoprecipitated samples (ii). PLP pull-down of HEK293 lysates immunoblotted with GFP antibody shows loss of polyproline binding of GFP-Pfn1-H133S (iii). (**B**) GFP-immunoblot of lysates from MDA-MB-231 clones shows comparable levels of exogenous Pfn1 expression (upper blot). Reprobing the blot with Pfn1 antibody shows similar levels of endogenous Pfn1 expression between the various cell lines (lower blot). (**C**) × 10 phase-contrast images show clustering of GFP-Pfn1-expressing MDA-MB-231 cells (bar – 100 *μ*m). (**D**) × 20 phase-contrast images show dramatically increased spreading for GFP-Pfn1-expressing cells compared to the other cell lines (bar – 50 *μ*m). (**E**) Immunoblot of cell lysates shows no evidence of E-cadherin expression in any of the engineered MDA-MB-231 cell lines (HMEC lysate in lane 1 serves as a positive control for E-cadherin blot).

As a complementary approach to gene silencing, we next adopted overexpression of GFP-Pfn1 in MDA-MB-231 cells as previously accomplished in BT474 breast cancer cells (Roy and Jacobson, 2004). Several different point mutants of Pfn1 that are defective in binding specifically to actin (H119E, R74E) and polyproline (H133S, W3A, W3N) have been reported in the literature (Bjorkegren-Sjogren *et al*, 1997; Suetsugu *et al*, 1999; Lambrechts *et al*, 2006). To selectively modulate Pfn1's interactions with actin and proline-rich proteins, we adopted overexpression of H119E and H133S mutants of Pfn1 (also GFP-fused), respectively. To initially confirm that fusing GFP to Pfn1 mutants still preserves their selective loss of functions, we transiently expressed each of these Pfn1 constructs in HEK293 cells. Co-immunoprecipitation (Figure 3A i–ii) and polyproline pull-down assays (Figure 3A-iii) confirmed loss of actin and polyproline binding specifically for GFP-Pfn1-H119E and GFP-Pfn1-H133S, respectively.

We next engineered MDA-MB-231 cells to stably express either GFP (control) or different Pfn1 constructs as indicated above. GFP-immunoblot of cell lysates showed comparable expression levels of different forms of exogenous Pfn1 in MDA-MB-231 sublines (Figure 3B). There was no appreciable change in endogenous Pfn1 expression between the various cell lines (Figure 3B). Quantitative immunoblots using known amounts of recombinant Pfn1 and GFP as standards showed that the expression level of GFP-Pfn1 or its mutants is about 60% of that of endogenous Pfn1 (data not shown).

Morphological examination showed that GFP-Pfn1-expressing cells tend to form significant clusters in normal culture (Figure 3C), and are also much more spread-out compared to other engineered MDA-MB-231 cell lines (Figure 3D). Since neither of the mutant cell lines exhibits increased spreading, it implies that interactions with both actin and proline-rich ligands are necessary for Pfn1-induced enhancement in cell spreading. Although clustered morphology is a typical epithelioid feature, our immunoblot data showed that Pfn1-overexpressing cells do not re-express epithelial marker E-cadherin (Figure 3E). Therefore, Pfn1 overexpression does not appear to induce complete epithelial reversion of MDA-MB-231 cells, and clustered morphology of Pfn1-overexpressing cells is likely to be due to their decreased ability to disseminate in culture.

### Pfn1 overexpression reorganises actin cytoskeleton and upregulates FAs in MDA-MB-231 cells

**Figure 4:**
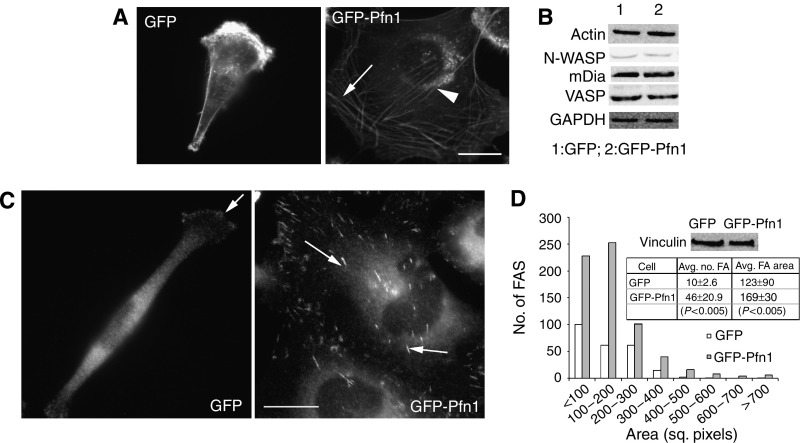
Pfn1 overexpression affects actin cytoskeleton and focal adhesion in MDA-MB-231 cells. (**A**) Phalloidin staining shows prominent actin cables that are organised as peripheral F-actin belt (arrow) as well as in a stress-fibre-like fashion (arrowhead) in GFP-Pfn1-expressing cells, while control GFP-expressing cells lack this feature (bar – 20 *μ*m). (**B**) Immunoblots of total cell lysates show comparable expression levels of actin, VASP, mDia1 and N-WASP between the control and Pfn1-overexpressing cells (GAPDH blot serves as the loading control). (**C**) Vinculin immunostaining show that GFP-Pfn1-expressing cells develop larger and more numerous FA plaques (arrows) than GFP-expressing cells (bar – 20 *μ*m). (**D**) A histogram summarises the difference in the number and the size of FAs between GFP- and GFP-Pfn1-expressing cells (19 cells of each type were analysed from at least four independent experiments). The immunoblot in the inset shows comparable levels of vinculin expression between these two cell types.

Previous studies showed diverse cellular effects of overexpressed or microinjected Pfn1, where in some cases an increase in the overall F-actin content was noticed (Finkel *et al*, 1994) and in others depolymerisation of existing actin filaments occurred (as could be expected from its G-actin-sequestering property – Balasubramanian *et al*, 1994). This demonstrates the complexity and cell-type specificity of Pfn1's action on actin cytoskeleton. In our case, phalloidin-staining showed that both parental (data not shown) and GFP-expressing MDA-MB-231 cells typically exhibit cortical F-actin staining with a stronger bias at the leading edge (Figure 4A). A distinguishing feature of GFP-Pfn1 expressers is the formation of long actin cables that are organised as peripheral F-actin belt (arrow) as well as in a stress-fibre-like fashion (arrowhead – Figure 4A). Immunoblot analyses of total cell lysates confirmed that there are no marked differences in the expression levels of either actin or other known major promoters of actin polymerisation, such as VASP, N-WASP and mDia1, between these two cell lines (Figure 4B). These data therefore demonstrate that even a very moderate level of Pfn1 overexpression can cause a marked change in the cytoskeletal organisation of MDA-MB-231 cells, and it is most likely via direct modulation of actin polymerisation and/or bundling of actin filaments. Furthermore, we observed that Pfn1 overexpression significantly increases the number and size of vinculin-positive FA plaques without appreciably affecting the total expression level of vinculin (Figures 4C–D). These data demonstrate that Pfn1 overexpression upregulates FAs in MDA-MB-231 cells.

### A functional actin-binding site is required for Pfn1 to suppress MDA-MB-231 cell motility

**Figure 5:**
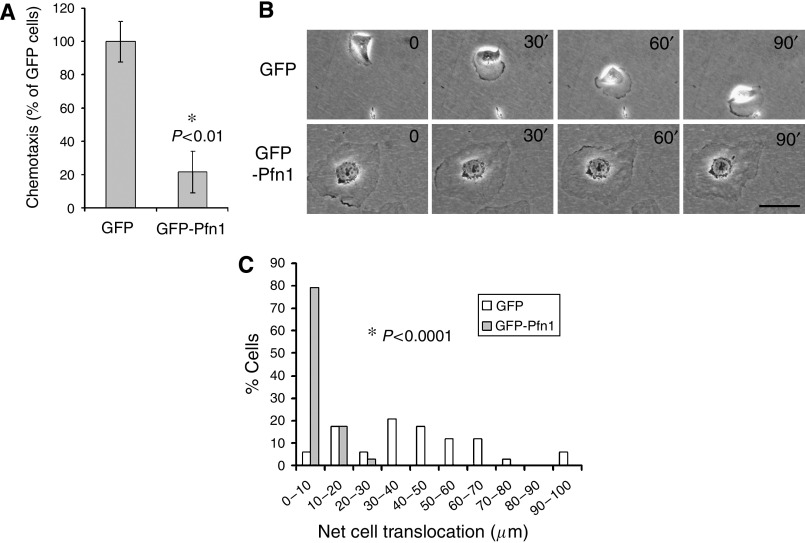
Effects of Pfn1 over expression on MDA-MB-231 cell motility. (**A**) Transwell experiments reveal that overexpression of Pfn1 significantly inhibits chemotactic migration of MDA-MB-231 cells (*N*=3 experiments). (**B**–**C**) Time-lapse imaging data of GFP and GFP-Pfn1 expressers show that Pfn1 overexpression dramatically inhibits cell translocation (**B**; bar – 50 *μ*m). A histogram (**C**) summarises the distribution of % cells *vs* net translocation distance (*n*=34 cells of each cell type pooled from two independent experiments).

To determine the effect of overexpression of Pfn1 in cell motility, we first performed transwell migration experiments which showed that overexpression of GFP-Pfn1 suppresses chemotaxis of MDA-MB-231 cells by nearly 80% (Figure 5A). Since the ability of cells to migrate through the pores in a transwell filter may be affected by their size (a potential issue for GFP-Pfn1-expressing cells), we also performed single-cell motility assay to confirm our transwell results. These experiments showed that GFP-expressers are able to randomly migrate with significant cell translocation (Figure 5B). By contrast, GFP-Pfn1-expressing cells fail to generate spatial asymmetry (almost maintains the same shape) and translocate during the course of observation (Figure 5B). A histogram in Figure 5C summarises the time-lapse data from two independent experiments which shows that the net distance translocated by GFP-expressing cells (41±23.3 *μ*m) is almost six-fold higher than that calculated for GFP-Pfn1 expressers (7±5.7 *μ*m).

**Figure 6:**
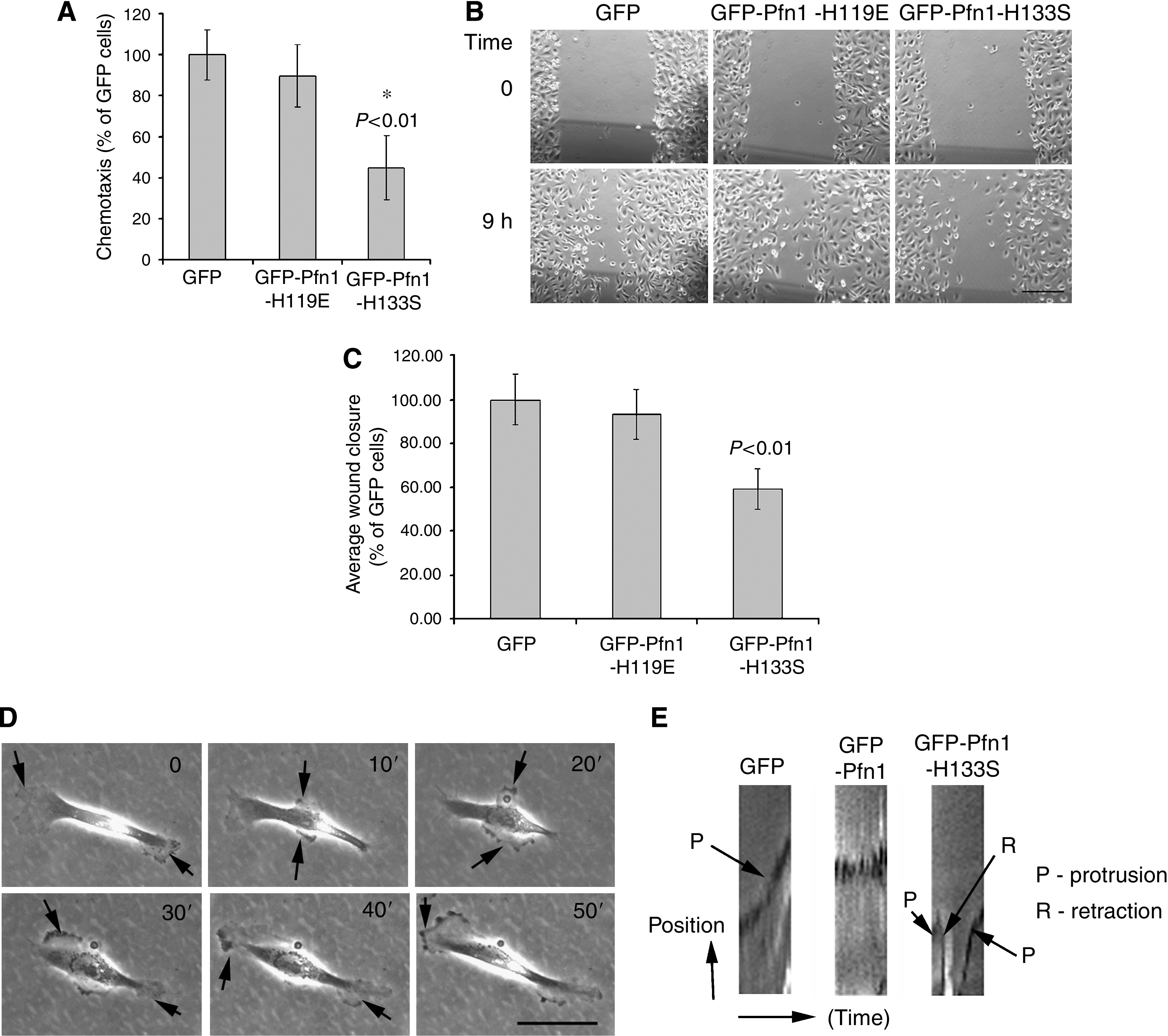
Effects of overexpression of Pfn1 mutants on MDA-MB-231 cell motility. (**A**) Transwell experiments show that overexpression of H133S, but not H119E mutant of Pfn1 inhibits chemotaxis of MDA-MB-231 cells (*N*=3 experiments). (**B**–**C**) Representative images of wound-healing experiments (**B**; bar – 200 *μ*m) and a bar graph (**C**) further illustrate the inhibitory effect of GFP-Pfn1-H133S on cell migration (*N*=4 experiments). (**D**) Time-lapse imaging shows GFP-Pfn1-H133S-expressing cells generate multiple, randomly directed protrusions (arrow; bar – 50 *μ*m). (**E**) Representative kymographs illustrate differences in lamellipodial behaviour between the various cell lines.

We next assessed the effect of overexpression of H119E and H133S mutants of Pfn1 on MDA-MB-231 cell motility. Data from transwell experiments showed that overexpression of GFP-Pfn1-H133S suppresses chemotaxis of MDA-MB-231 cells by 55%, whereas no significant difference in chemotaxis is observed between GFP and GFP-Pfn1-H119E-expressing cells (Figure 6A). Further confirmation of these results came from wound-healing experiments which showed a similar 40% inhibition in motility induced by GFP-Pfn1-H133S overexpression with GFP-Pfn1-H119E expression having no effect at all (Figures 6B, C). These results were verified with two different clones of each of our mutant cell lines and thus our findings are not clone-specific effects. Overall, these data thus demonstrate that a functional actin-binding site is required for overexpressed Pfn1 to suppress MDA-MB-231 cell motility. Although overexpression of both functional Pfn1 and its H133S mutant suppress cell migration, these two cell lines behave very different as revealed from time-lapse imaging data. In contrast to GFP-Pfn1 expressers, cells expressing GFP-Pfn1-H133S are highly dynamic, generate multiple and randomly directed protrusions throughout the period of observation (Figure 6D). We found that net distance translocated by GFP-Pfn1-H133S expressers (19.7±16.1 *μ*m; *n*=44 cells pooled from two experiments) is still almost two-fold less than the corresponding value for GFP-expressing cells; this finding is in good agreement with our previous transwell and wound-healing data. Since one of the important functions of Pfn1 is to regulate actin polymerisation during cell protrusion, we next compared the lamellipodial dynamics of GFP, GFP-Pfn1, GFP-Pfn1-H133S expressers from their kymographs in order to obtain further insight on Pfn1-induced change in cell motility. As shown in Figure 6E, GFP-expressing cells, in general, tend to maintain relatively persistent protrusion as indicated by a steady positive slope of the kymograph. By contrast, GFP-Pfn1 expressers display nearly flat kymographs with minor oscillations of cell periphery thus indicating suppressed lamellipodial activity. Interestingly, GFP-Pfn1-H133S expressers show prominent protrusive activity; however, these protrusions often undergo rapid retractions (indicated by a negative slope of the kymograph), and are hence much less stable. Overall, these data demonstrate that perturbing Pfn1 alters the lamellipodial dynamics of MDA-MB-231 cells.

### Pfn1 is a negative regulator of the invasiveness of MDA-MB-231 cells

**Figure 7:**
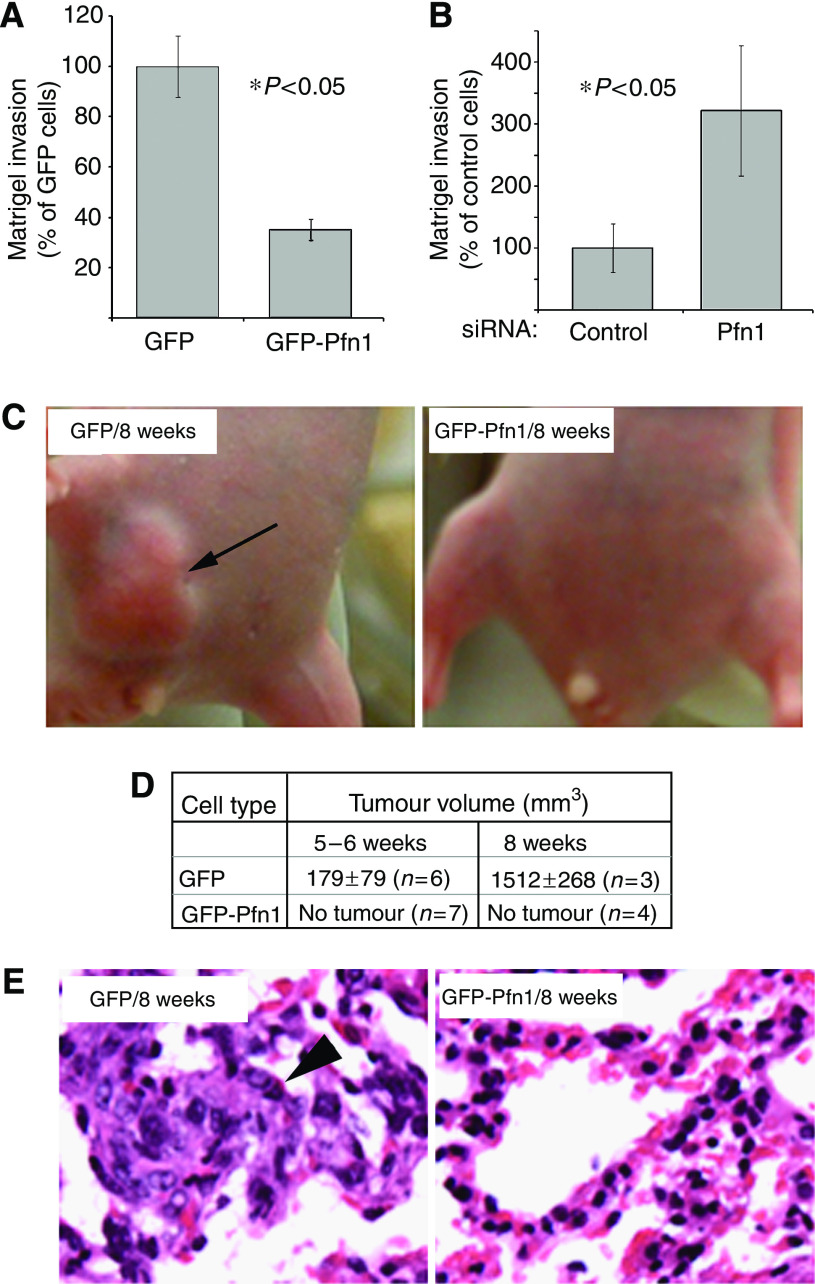
Perturbing Pfn1 overexpression alters matrigel invasiveness, orthotopic tumorigenicity and micro-metastasis of MDA-MB-231 cells. (**A**–**B**) Overexpression (**A**) and silencing Pfn1 expression (**B**) decreases and increases matrigel invasiveness of MDA-MB-231 cells, respectively (*n*=3 experiments). (**C**) Orthotopic inoculation of GFP-expressing MDA-MB-213 cells produce large tumour (arrow) in nude mice (left panel), while Pfn1 overexpressing cells fail to do so (right panel). (**D**) A summary of tumour-burden data at the time of killing. (**E**) Haematoxylin and eosin staining of lung tissue harvested at 8-week time point shows significant micro-metastasis (exhibit typical pleomorphism and higher nuclear content – arrowhead) in mice injected with GFP expressers (left panel), while lung histology appears completely normal in those injected with GFP-Pfn1 expressers (right panel).

To better mimic tumour cell invasion through ECM as occurs *in vivo*, we next compared matrigel invasiveness of GFP- and GFP-expressing MDA-MB-231 cells in response to a chemotactic gradient. Our data showed that overexpression of GFP-Pfn1 dramatically suppresses the matrigel invasiveness of MDA-MB-231 cells by nearly four-fold (Figure 7A). Even though these invasion experiments were carried out in a transwell-based chamber where the pore size could be a potential issue for larger GFP-Pfn1-expressing cells, our finding is valid for two reasons. First, we normalised the invasion readout by the number of transmigrating cells that was scored in the control chamber. Second, there was absolutely no difference in the number of transmigrating cells between the two groups at the 24-h time point of evaluation (long enough to allow all of the seeded cells to transmigrate). Consistent with our overexpression data, we found that cell invasiveness increases dramatically by nearly three-fold when Pfn1 expression is suppressed (Figure 7B). Overall, these data prove that Pfn1 negatively regulates the intrinsic invasiveness of MDA-MB-231 cells.

### Pfn1 overexpression limits orthotopic tumorigenicity and metastasis of MDA-MB-231 cells

The real measure of tumour invasiveness for dissemination is the ability to form metastases in animals from an orthotopic tumour site. *In vivo* effect of Pfn1 overexpression on mammary tumour cells has been examined for only one cell line (CAL51) so far where suppression of tumorigenicity was observed (Janke *et al*, 2000; Wittenmayer *et al*, 2004). However, these studies were performed in a non-orthotopic model system. Since microenvironment has a critical influence on tumour progression, we extended our line of inquiry to MDA-MB-231 cells in an orthotopic xenograft model system where we injected either GFP (control) or GFP-Pfn1-expressing cells into the inguinal mammary fat pad of nude mice. The GFP-expressing cells formed visible tumours as expected; however, tumour formation was completely suppressed in mice injected with GFP-Pfn1-expressing cells (Figure 7C). The tumour burden data at the time of killing is summarised in a tabular form in panel D. Histology of lungs harvested from animals injected with GFP-expressing cells revealed significant micro-metastasis when evaluated at the 8-week time point, whereas the same for animals bearing GFP-Pfn1 expressers appeared completely normal (Figure 7E). Taken together, these data demonstrate that Pfn1 overexpression suppresses orthotopic tumorigenicity and micro-metastasis of breast cancer cells.

## DISCUSSION

While it has long been known that Pfn1 modulates the actin cytoskeleton, its overall regulatory role was not well defined. Since Pfn1 has been traditionally viewed as a molecular player required for cell migration, it is not at all clear why Pfn1 expression is downregulated in various forms of invasive cancer cells. In the present study, we evaluate for the first time how perturbing Pfn1 affects the motility and invasiveness of breast cancer cells as well as their normal counterparts. Several novel findings are reported in this study. First, we demonstrate that near complete loss of Pfn1 expression actually confers increased motility and invasiveness to breast cancer cells. Conversely, overexpression of Pfn1 suppresses motility and invasiveness of breast cancer cells. Taken together, these observations suggest for the first time that Pfn1 is a negative regulator of mammary carcinoma aggressiveness. Second, we find that silencing Pfn1 expression in HMEC leads to downregulation of both cell–cell and cell–matrix adhesions, increased motility and dramatic scattering. Since these cellular changes are precursor to tumour cell dissemination *in vivo*, our findings for the first time provide a possible insight on why Pfn1 expression has to be downregulated in breast cancer cells. Third, the present study is the first demonstration of the *in vivo* effect of Pfn1 overexpression on breast cancer cells in an orthotopic setting. Our finding that Pfn1 overexpression suppresses orthotopic tumorigenicity of MDA-MB-231 cells reproduces previous results reported for another breast cancer cell line in subcutaneous xenograft models (Janke *et al*, 2000; Wittenmayer *et al*, 2004), and thus provides a further supportive evidence for the tumour-suppressive property of Pfn1 in breast cancer.

Gain of motility-related function after Pfn1 depletion has been unprecedented in the literature. Although further mechanistic studies are needed to elucidate why Pfn1 depletion increases motility in breast cancer cells, yet dramatically inhibits motility of other cell types, such as vascular endothelial cells (Ding *et al*, 2006), certain speculations may be appropriate. First, whether Pfn1 would inhibit or facilitate actin polymerisation depends on its concentration relative to G-actin and uncapped barbed ends of actin filaments. These parameters are also regulated by other ABPs, the cellular concentrations of which vary between different cell types. Because of this complex nature of its action on actin cytoskeleton, Pfn1 can have either concentration and/or cell-type dependent effect on cell motility. Given that the concentration of residual Pfn1 after gene silencing could also vary between different cell systems, a direct comparison can sometimes be difficult.

Second, disruption of actin filaments is a known feature following oncogene-induced cell transformation (Pawlak and Helfman, 2001). Clearly, literature has documented evidence of elevated expression of thymosin *β*4 (a G-actin-sequestering protein that inhibits actin polymerisation) and loss of tropomyosin (an F-actin-stabilizing protein) correlating with increased aggressiveness of carcinoma cells (Franzen *et al*, 1996; Kobayashi *et al*, 2002; Wang *et al*, 2004). In fast migrating tumour cells, a moderate decrease in cellular F-actin level can potentially enhance cell motility by either increasing the availability of G-actin for actin-treadmilling, and/or decreasing the overall rigidity of cell cortex (enhances membrane deformability). Thus, it is conceivable that by lowering the overall F-actin content in MDA-MB-231 cells, Pfn1 depletion may create a similar cytoskeletal background and favour cell motility provided (1) other F-actin-dependent processes that are otherwise critical for normal cell motility, such as contractility, becomes less important, and (2) Pfn1 becomes dispensable in the case of tumour cells. In general, highly invasive tumour cells like MDA-MB-231 are weakly adherent in nature and thus, contractility may be of much lesser significance in the context of motility of these cells when compared to some of the more strongly adherent cell types, such as vascular endothelial cells. A noteworthy point here is that in our previous study we observed a dramatic decrease in actin stress-fibres and FAs (the molecular machineries for generating cell contractility) in vascular endothelial cells after silencing Pfn1, which could partly account for their translocation defect (Ding *et al*, 2006). Among its diverse functions, Pfn1 has been most heavily implicated in actin-based cell protrusion. We previously found that vascular endothelial cells lacking Pfn1 are drastically impaired in generating polarised protrusion (Ding *et al*, 2006). In the case of MDA-MB-231 cells, although we noticed a somewhat decrease in the overall flare of protrusion after Pfn1 depletion, polarised protrusion at the leading edge was still evident. It was previously implied that Pfn1 facilitates, but is not absolutely critical for the canonical WASP-Arp2/3-mediated, actin-based protrusions in migrating cells (Loisel *et al*, 1999). Since WASP-family proteins, Arp-2/3 as well as their upstream regulators (Rac1, Cdc42) are known to be overexpressed in several invasive cancers including breast cancer (Sahai, 2005), protrusion of tumour cells may be much less sensitive to loss of Pfn1 expression compared to vascular endothelial cells. Also, suppression of certain protrusions, which are unproductive for forward movement, such as those arising at the lateral edge of the cell, leads to better spatial asymmetry of cells (a key requirement for directional migration). Thus, it is possible that by lowering the overall flare of protrusion, depletion of Pfn1 may actually facilitate this process and enhance directional motility. This is not unreasonable since we observed exactly opposite phenomena in the case of Pfn1-overexpressing cells which display aberrant protrusions in all directions, fail to generate spontaneous spatial asymmetry and are significantly impaired in translocation (also reproduces our previous finding in BT474 cells; Roy and Jacobson, 2004). Excessive lamellipodial protrusion observed in these cells could be a combined result of (1) Pfn1-induced stimulation of actin polymerisation at the leading edge, (2) better stabilisation of protrusions because of upregulated FAs and (3) a possible defect in cell retraction due to increased adhesion.

Another interesting and novel finding is that between the two Pfn1 mutants, overexpression of only poly-proline binding-deficient mutant (has functional actin binding) suppresses MDA-MB-231 cell motility. Since we showed that loss of Pfn1 expression actually enhances motility, our findings should not be interpreted as that Pfn1's interaction with proline-rich ligands is required for MDA-MB-231 cell motility. Our data rather suggest that actin binding is necessary for Pfn1-induced suppression of breast cancer cell motility. Because of differences in both morphology and behaviour in time-lapse assays between GFP-Pfn1 and GFP-Pfn1-H133S expressers, it appears that there might be some unique differences in the underlying mechanisms of inhibition of cell motility induced by these two constructs. Multiple and randomly directed protrusion observed in GFP-Pfn1-H133S expressers partially resonates with a recent finding that disrupting polyproline interaction of Pfn1 promotes neurite elongation in PC12 cells (Lambrechts *et al*, 2006). Randomness of protrusions (impedes motility in one particular direction) could be the one of the main reasons why directional motility is inhibited by the H133S construct.

Although our matrigel invasion data successfully demonstrate that Pfn1 is a negative regulator of the intrinsic invasiveness MDA-MB-231 breast cancer cells, because of heterogeneity of tumour cells, it will be valuable to extend these studies to both primary cancer cells and other invasive/noninvasive breast cancer cell lines with different endogenous levels of Pfn1. Importantly, we will have to address key mechanistic questions such as whether perturbing Pfn1 affects the secretion of matrix-degrading proteolytic enzymes (an integral aspect of cell invasion) or primes tumour cells to preferentially adapt to any special kind of motility (mesenchymal *vs* ameboid) during the process of invasion. To better mimic the *in vivo* process of tumour cell invasion, these future studies will need to utilise a tumour-microenvironment model including stromal cells (fibroblasts, macrophages) in the culture. Finally, even though our *in vivo* studies show lack of micro-metastasis of Pfn1-expressing cells, we cannot resolve whether this is due to failure of these cells to disseminate via reduced migration and invasion (as suggested by *in vitro* studies) or lack of tumour formation. Future studies are needed to determine whether targeted overexpression of Pfn1 in preformed tumours slows down distant metastasis. If true, perturbing Pfn1 could prove to be a good molecular strategy for limiting aggressiveness of breast cancer cells.
